# Wow, I cannot stop: a concentration on vocabulary learning via instagram and its effects on informal digital learning of english, technostress, and on-line engagement

**DOI:** 10.1186/s40359-023-01503-w

**Published:** 2024-01-02

**Authors:** Ali Rashed Ibraheam Almohesh, Jinan Abdulaziz Hamad Altamimi

**Affiliations:** Department of Arabic Language, College of Education in Al-Kharj, Prince Sattam Bin Abdulaziz University, Al-Kharj, Saudi Arabia

**Keywords:** Vocabulary learning, Instagram application, Informal digital Learning, Technostress, On-line engagement, EFL learners

## Abstract

The proliferation of social media in contemporary society presents a compelling impetus for scholars to explore the untapped capabilities of social networking platforms with the aim of enhancing educational practices. Despite the notable impact of social media on the acquisition of second or foreign languages, there is a scarcity of empirical research investigating the influence of Instagram feed-based activities on the development of vocabulary in language learning at under-intermediate level. In order to address this research gap, the present study aimed to investigate the impact of Instagram feed-based activities on the acquisition of vocabulary among individuals studying English as a foreign language (EFL). As a result, a cohort of 79 intermediate EFL learners was subjected to random assignment, leading to the formation of both experimental and control groups. The participants in the control group were provided with conventional online teaching using webinar platforms. The participants in the experimental group, on the other hand, were instructed via the use of webinar platforms and were required to participate in exercises that were based on Instagram feeds respectively. The results of the data analysis using one-way Multivariate Analysis of Variance (MANOVA) revealed that the learners in the experimental group (EG) demonstrated superior performance compared to their counterparts in the control group (CG). The findings underscored the notable impact of using Instagram feed-based activities in promoting the acquisition of vocabulary knowledge. Additionally, it was shown that EFL learners had favorable views towards the use of Instagram Feed-based Tasks for the purpose of learning vocabulary. Due to treatment, EG achieved higher state in informal digital learning of English and on-line engagement. Moreover, they felt less technostress after treatment. The findings of this research have potential consequences for language learners, instructors, curriculum designers, and policy makers. Specifically, they suggest that incorporating Instagram feed-based activities into language learning and teaching may provide valuable chances for further practice.

**Supplementary Information:**

The online version contains supplementary material available at 10.1186/s40359-023-01503-w.

## Introduction

The advent of the Internet has inaugurated a novel epoch for technology that enables the facilitation of communication and the sharing of information. This incident acted as the catalyst for the first phases of social media’s growth. Social media originated due to the need for a portable, affordable, and quickly accessible information and communication technology tool. Obtaining prompt and unmediated feedback via the use of social media is achievable [1]. According to [2], social media refers to internet-based applications that enable the sharing of pictures (e.g., Instagram), organization of information (e.g., Pinterest), transmission of photos or videos (e.g., Skype), exchange of instant messages (e.g., WhatsApp), or a combination of these functions (e.g., Facebook). According to [3], social media can be classified into the following categories: (1) social networks (such as Facebook and LinkedIn); (2) bookmarking sites (such as Delicious and StumbleUpon); (3) social news (such as Digg and Reddit); (4) media sharing platforms (such as Instagram, YouTube, and Flickr); (5) microblogging (such as Twitter); and (6) blogging, specifically comments and forums. Due to its affordability, convenient portability, and widespread availability, social media is swiftly gaining widespread use worldwide. The widespread popularity of these platforms has led many educators to investigate the educational opportunities they provide, and language learning is no exception to this trend.

Language instruction was given a much-needed boost by the advent of social media platforms, which fostered a more interactive and collaborative learning environment [4–6]. Moreover, social media platforms are adaptable, meaning they may be utilized in a variety of settings for learning (e.g., K-12 and higher education) [5]. Technology for portable gadgets has advanced rapidly in recent years. The original intent of mobile phones was for spoken communication, but new developments in mobile device technology have defined new roles for these devices. Users may do things like conduct research, send and receive emails, read e-books, and make purchases online regardless of their location or the time of day [3]. Mobile assisted language learning (MALL) is a subfield of m-Learning and a subset of computer assisted language learning (CALL) [7, 8] that refers to the incorporation of mobile device technology into instruction and education. [9] state that mobile technologies’ benefits include being adaptable, inexpensive, compact, and simple to use.

The digital environment, on the other hand, presents a significant challenge: the question of how to motivate students to participate and interact in meaningful ways. The level of student engagement, which may be described as the degree to which students are actively involved in educational activities that result in the desired learning outcomes [10, 11], is an essential factor in ensuring the success of online learning. Better language teaching methods may be developed with the help of a more sophisticated knowledge of students’ levels of involvement in the classroom. Issues such as indirect social interactions, insufficient pupils and educators and peer interactions, and the necessity of pupils to acclimate to the online setting give the notion of pupil involvement in distance-learning programs a new dimension [4, 5, 9]. Furthermore, problems about persistence and efficiency usually arise, which makes it challenging to maintain consistent and meaningful student engagement [11].

Informal Digital Learning of English (IDLE) is an essential component that has a profound impact on student engagement. The idea of IDLE has similarities with other related concepts, such as online English learning and extramural English, in terms of its goals and instructional recommendations [12]. This emerging practice in EFL classrooms involves students independently engaging in language activities via non-academic digital platforms, driven by their own interests and desires rather than the requirements of the educational program [13]. Taking a similar path [14], provide evidence that IDLE is closely connected with intercultural competence and willingness to communicate in language learning. Although there has been a growing interest in using IDLE for language learning, the relationship between IDLE and student engagement has not been thoroughly examined. Hence, there is an urgent need for study on this element, specifically in regards to the level of active involvement of EFL students in their online learning. This kind of study has the capacity to provide valuable insights into the potential role of IDLE in enhancing learners’ commitment and active participation in virtual language learning experiences.

A widespread use of technology has resulted in the emergence of detrimental user experiences, such as technostress [15]. Originally used the word “technostress,” which he characterized as an “adaptive sickness” caused by an individual’s incapacity to effectively and healthily deal with advances in computer technology. Technostress, according to the research of [16], may be thought of as any negative effect that technology has on an individual’s mental state, behavior, or physiological systems. The use of computers in higher education is increasing, yet despite the fact that technostress has been studied in other contexts, it has not yet been examined in this area. Burnout, depression, and exhaustion are only some of the long-term health issues that have been related to technostress [17]. Working in a technologically demanding environment is not automatically associated with a negative technostress outcome. Fortunately, there are ways to lessen these adverse impacts of technostress [18]. Technostress must be addressed not just when its negative consequences have become evident, but also during its whole life cycle.

Technostress has been investigated for how it affects individuals’ openness to new technology. Technostress is a consequence of the increasing use of technology, resulting in detrimental effects on users [19]. Was the originator of the term technostress, which he characterized as a maladaptive condition resulting from a failure to effectively and healthily manage the challenges posed by developing computer technology. Technostress refers to the negative effects on an individual’s mental moods, behaviors, or physiological processes that are caused by technology, either directly or indirectly [15]. According to [14] research, teachers’ tendency to employ technology solutions decreases when they experience technostress. In addition [17], found that technostress mediates negatively between students’ estimations of the value of digital textbooks and their intentions to buy them. When people are under a lot of stress from technology, they are less likely to let their appreciation of its practicality affect their decision to use it. Technostress and other unpleasant emotions were discovered to have an effect on people’s attitudes toward new technology, according to the findings of [18]. It is conceivable for various persons to come at different conclusions about the link between perceived norms and behavioral intention [19]. This is due to the fact that the degrees of technostress that individuals experience vary based on the traits that they possess of themselves. Similarly [20], emphasized that using a Portfolio as a means of expediting language evaluation might reduce Techno-Stress among EFL learners and enhance their Test Taking Skills, Buoyancy, and Language Achievement.

Multi-faceted in its conceptualization, engagement is defined as the degree to which an individual is actively involved in and committed to a particular endeavor [21]. Student engagement involves goals, community, and achievement in the classroom [22]. For example, according to [23], student engagement may be defined as learners’ eagerness, motivation, and drive to take an active role while achieving success in their own learning. Early studies concentrated primarily on the behavioral dimension, which takes into account one’s outlook and approach to learning but leaves out one’s perceptions of and reactions to any difficulties encountered while learning [24]. After examining the affective and behavioral components, they moved on to the cognitive one [25]. Therefore, we now know that student involvement has four components: actions, emotions, thoughts, and relationships [26].

Positive views and behaviors toward educational endeavors are what constitute active participation, whereas negative ones are avoided [27]. When students are emotionally engaged, they are responding positively to their instructors, peers, and course material [22]. To succeed in different types of learning environments, cognitive engagement highlights the significance of self-regulation, learning techniques, and cognitive efforts [28]. Finally, social engagement is the propensity for students to communicate with their teachers, peers, and the material itself [29] and with one another. Each of these factors is special in its own way and has a major impact on students’ motivation to learn [30]. In order to create a classroom climate that encourages students to actively participate and thrive, teachers must have a deep and nuanced understanding of the many factors that contribute to student engagement.

Previous studies have attempted to study the possible antecedent of students’ participation in online learning. For example [31], tended to address the function of a student-facing social learning analytics tool in affecting student engagement in online collaborative writing. This was done by focusing on the role of a student-facing social learning analytics tool. The researchers used a mixed-method approach, and the results showed that the student-facing social learning analytics tool had a significant impact on the amount of time learners spent actively participating in online activities [27]. Explored the influence of attitudes toward learning in determining the involvement of students in their online learning, drawing on the self-determination theory as their theoretical foundation. The authors observed that the students’ views about online learning might have a significant influence on the level of online engagement that students experienced.

The assessment of the existing research reveals that the empirical evidence on online involvement has explored several aspects that have contributed to the issues under consideration. However, the impact of vocabulary acquisition via Instagram on language learning and teaching, as well as its influence on informal digital English learning, technostress, online engagement, and language proficiency, remains untapped. In order to fill this void in research, the following questions are posed:

1. Does vocabulary learning via Instagram influence the state of informal digital learning.
2. Does vocabulary learning via Instagram influence the state of Technostress among EFL Learners?
3. Does vocabulary learning via Instagram influence the state of On-Line Engagement among EFL Learners?

## Theoretical framework

The concept of social presence, which originates from the idea of immediacy [32], is also conceptually tied to the engagement of students in online education. How well people express themselves and connect with others in a virtual space is a measure of their “social presence” [32,33]. Posits that students will be more invested in their coursework if they experience a higher degree of social presence in their online interactions with lecturers and classmates. A favorable correlation between students’ social presence and their participation in online courses may therefore be hypothesized on the basis of this theory. In addition, connectivism serves as the theoretical foundation for this investigation. Connectivism is a modern paradigm of learning that advocates for pupils to integrate different types of knowledge and information. It recognizes that technological tools are essential to education and that students’ ongoing connectivity opens up avenues for educational autonomy. Remote learning [34] gave rise to connectivism, which characterized the course’s digital modes of communication. Connectivism, in other words, is defined and supports skill learning via digital connections and social media [34]. Finally, the concept of taking language learning outside the classroom may be conceptually related to IDLE’s influence on student involvement [35]. Since online students are exposed to the target language for longer periods of time, they may acquire a higher level of competency in that language over time.

## Methodology

The present investigation is quantitative in scope and employs a quasi-experimental design consisting of a pre-test and a post-test. The actions that were done are described in further depth in the following paragraphs.

### Participants

Based on the outcomes of the Oxford Quick Placement Test, a random sample of 84 individuals was selected from a larger group of 136 first-year EFL students studying at the Prince Sattam Bin Abdulaziz University, Saudi Arabia. There were 44 females and 35 males in the sample. The participants’ levels of English language competence were determined to be intermediate based on the results of the Oxford Quick Placement Test. In addition, they did not participate in any additional English lessons over the course of the study endeavor. Because of this, the starting point of the research found that the participants’ levels of English language ability were comparable to one another. They ranged in age from 17 to 23, and came from a variety of socioeconomic and cultural backgrounds. In EG there were 41 students, while in CG there were 38 students. The students were required to attend sixteen sessions of an English grammar class during the first semester of the academic year, as outlined in their respective syllabi. The students were fully aware that participation in this study was entirely optional, and they provided their informed permission to be a part of this research endeavor.

### Materials

#### Oxford quick placement test (OQPT)

The participants’ English competence was evaluated using the OQPT (See Additional File 1). There is a wide range of possible results on the OQPT (from 0.1 to 0.9), but a score of between 0.4 and 0.6 indicates an intermediate level of English ability. In this research, the OQPT had a reliability of 0.91.

#### English vocabulary pre-test and post-test

In accordance with the contents of the recommended resources, a custom exam was developed by the researchers (Key Words for Fluency, Intermediate) (See Additional File 2). The 40 questions on this exam are split evenly between multiple-choice, short-answer, true-false, closed test formats. Expert judgment was used to examine the items for both external and internal consistency. Three psychometricians and six EFL faculty members were consulted for their opinions on the items’ quality. Their feedback led to certain changes being made. The test’s test-retest reliability was then evaluated by administering it to 31 college students, representative of the study’s target demographic. The same test was given to the same individual again 2 months later to evaluate the reliability of the findings across time. Test-retest reliability was determined to be good (r = .87, p 0.05) based on the findings of the Pearson correlation coefficient.

#### Informal digital learning of english scale

The IDLES, which was first established by [36], was customized for the purposes of this research in order to evaluate the participants’ informal digital English learning. It includes form-focused activities (3 items), game-based activities (2 items), receptive activities (4 items), and productive activities (4 items). Each of these subscales measures receptive and productive activities. On a scale that was similar to the one used by Likert; the participants rated how often they participated in these activities on a scale that ranged from 1 (never) to 5 (very often). Subsequently, Cronbach’s alpha was used to assess the internal consistency, yielding a score of 0.855.

#### Technostress

The buoyancy of the EFL learners was assessed using the Techno-stress Scale (T-S S) by [23]. The eight distinct items were taken into consideration while developing this scale. It used a Likert scale with a point value of 1, representing significant disagreement, and a point value of 5, representing strong agreement. In addition, T-S S dependability was shown to be within acceptable levels (between 0.79 and 0.83).

#### On-Line engagement

In this research, an instrument that was previously created by [37] and consisted of 16 different questions was used to measure the level of involvement that online students had with their coursework. The survey featured a Likert scale with five points, and it consisted of four separate subscales: behavioral engagement (BE), cognitive engagement (CE), affective engagement (AE), and social engagement (SE). There were four items that were used to measure each subscale. The reliability of this scale, as assessed by Cronbach’s alpha, was deemed satisfactory (0.863).

#### Procedure

The present research used a quasi-experimental design in its first stages, with individuals randomized to groups according to non-random criteria. Initially, the students’ English competence was evaluated using OQPT. Seventy-nine participants were selected using the cut score (0.4–0.6) from the results they obtained on the Oxford Quick Placement Test (score range: 0.1–0.9). Students who scored higher (between 0.7 and 0.9), indicating stronger language competence, were not included in the research, whereas those with an intermediate level of English language proficiency were. In addition, the participants in this study were advised to refrain from taking any further English courses. A preliminary examination was performed before the therapy was given.

Following the pre-test, one of the researchers, who was also the teacher for all of the courses taken by either the EG or CG, took over the role of providing instruction. This research was conducted over the course of one academic semester, which consisted of sixteen sessions, in the year 2022. The students in the CG, which consisted of 38 students, were provided with consistent online education via the use of webinar platforms (Adobe connect). Learners in the EG (41 students), on the other hand, learn and practice vocabulary using a social website on Instagram that was established by the researchers beside their selected instruction, Adobe connect. This page on Instagram posted a variety of activities that were designed to teach and practice English vocabularies. These activities were based on the planned book (Key Words for Fluency) as well as the course curriculum. The introduction of each subject matter in this book was accomplished with the assistance of engaging postings that included voice, video, and photographs. Instagram allows users to transmit photographs, audios, and videos to one another, and it is accessible to both instructors and students. Moreover, the students have the opportunity to pose questions, respond to them, and get feedback. In order to prevent any discrepancy from occurring throughout the treatment, it was requested that the students who were a part of the EG not disclose the knowledge to their counterparts who were a part of the CG.

A post-test was administered at the conclusion of the semester (16 sessions), after all of the teaching had been finished. The purpose of the exam was to evaluate the accomplishments of the students in both the CG and EG, as well as to determine how successful the program had been. In order to guarantee the accuracy of the results, three different EFL instructors graded both the pre-test and the post-test. In the end, we took into consideration each student’s pre-test score as well as their post-test average. Due to the fact that all of the respondents met the requirements necessary to comprehend English, the questionnaire was written in English.

#### Data analysis

The MANOVA was carried out to evaluate the data. The primary objective of MANOVA is to ascertain if the independent variables, either individually or in conjunction with each other, have an influence on the dependent variables. For MANOVA to be applicable, the dependent variables must satisfy parametric criteria. Analyses of the associated assumptions were performed before the MANOVA analysis was run. The normality of the data, the size of the sample, the presence or absence of outliers, the linearity of the data, and the homogeneity of the regression were among these.

## Results

Prior to do any analysis, it was necessary to investigate if parametric or non-parametric tests must be used. Thus, normality distribution of the data was checked through running the Kolmogorov–Smirnov test (KST). After running this test, it was revealed the data are normal (p > .05); thus, using MANOVA was safe. Table 1 shows the descriptive statistics of both EG and CG on the posttests of English Vocabulary (EV), Informal Digital Learning of English Scale (IDLES), Technostress, and On-Line Engagement (OLE).

**Table 1:** Descriptive Statistics of the Posttests of EG And CG on EV, IDLE, Technostress, and OLE

	Groups	Mean	Std. Deviation	N
EV Post	EG	37.0244	5.21291	41
	CG	29.4737	6.77729	38
	Total	33.3924	7.08088	79
IDLE Post	EG	49.3171	11.26596	41
	CG	29.0526	10.02954	38
	Total	39.5696	14.71845	79
Techno Post	EG	21.7073	7.48079	41
	CG	34.4474	5.04947	38
	Total	27.8354	9.04608	79
OLE Post	EG	52.3171	14.66363	41
	CG	36.0000	12.05169	38
	Total	44.4684	15.69978	79

Table 1 provides the mean and standard deviation for the four different dependent variables, which have been split by the independent variable. The mean scores of the EG on the EV, IDLE, Technostress, and OLE are 37.0244, 49.3171, 21.07073, and 52.3171, respectively. In addition, CG’s mean scores on the EV, IDLE, Technostress, and OLE are 29.4737, 29.0526, 34.4474, 36.0000, respectively. To check any potential differences among the two groups, MANOVA must be run (Table 2).

**Table 2:** MANOVA Results (The Performance of EG and CG on the Posttest of EV, IDLE, Technostress, and OLE

Effect		Value	F	Hypothesis df	Error df	Sig.	Partial Eta Squared
Intercept	Pillai’s Trace	0.336	8.856	4.000	70.000	0.000	0.336
	Wilks’ Lambda	0.664	8.856	4.000	70.000	0.000	0.336
	Hotelling’s Trace	0.506	8.856	4.000	70.000	0.000	0.336
	Roy’s Largest Root	0.506	8.856	4.000	70.000	0.000	0.336
Groups	Pillai’s Trace	0.793	67.173	4.000	70.000	0.000	0.793
	Wilks’ Lambda	0.207	67.173	4.000	70.000	0.000	0.793
	Hotelling’s Trace	3.838	67.173	4.000	70.000	0.000	0.793
	Roy’s Largest Root	3.838	67.173	4.000	70.000	0.000	0.793

The value of Wilk’s Lambda as shown in Table 2, is 0.000, revealing that there is a statistically significant difference (p < .05) between the EG and CG in terms of their posttest scores for EV, IDLE, Technostress, and OLE. It is thus essential to run the Table 3 to determine the causal relationship between the two groups and identify the specific dependent variable that contributed to the observed difference.

**Table 3:** Test of Between-Subjects Effects for EV, IDLE, Technostress, and OLE

Source	Dependent Variable	Type III Sum of Squares	df	Mean Square	F	Sig.	Partial Eta Squared
Corrected Model	EV Post	2941.119	5	588.224	44.281	0.000	0.752
	IDLE Post	10456.73^b^	5	2091.347	23.704	0.000	0.619
	Techno Post	4326.252	5	865.250	30.712	0.000	0.678
	OLE Post	7982.564	5	1596.513	10.366	0.000	0.415
Intercept	EV Post	344.455	1	344.455	25.931	0.000	0.262
	IDLE Post	202.058	1	202.058	2.290	0.135	0.030
	Techno Post	87.061	1	87.061	3.090	0.083	0.041
	OLE Post	800.505	1	800.505	5.198	0.026	0.066
EV Pre	EV Post	1741.373	1	1741.373	131.090	0.000	0.642
	IDLE Post	10.227	1	10.227	0.116	0.734	0.002
	Techno Post	7.451	1	7.451	0.264	0.609	0.004
	OLE Post	1.895	1	1.895	0.012	0.912	0.000
IDLE Pre	EV Post	3.796	1	3.796	0.286	0.595	0.004
	IDLE Post	2144.768	1	2144.768	24.309	0.000	0.250
	Techno Post	2.041	1	2.041	0.072	0.789	0.001
	OLE Post	31.330	1	31.330	0.203	0.653	0.003
Techno Pre	EV Post	0.237	1	0.237	0.018	0.894	0.000
	IDLE Post	128.747	1	128.747	1.459	0.231	0.020
	Techno Post	1073.403	1	1073.403	38.101	0.000	0.343
	OLE Post	9.985	1	9.985	0.065	0.800	0.001
OLE Pre	EV Post	0.184	1	0.184	0.014	0.907	0.000
	IDLE Post	32.852	1	32.852	0.372	0.544	0.005
	Techno Post	7.624	1	7.624	0.271	0.604	0.004
	OLE Post	2690.632	1	2690.632	17.470	0.000	0.193
Groups	EV Post	792.567	1	792.567	59.664	0.000	0.450
	IDLE Post	7371.939	1	7371.939	83.556	0.000	0.534
	Techno Post	2533.194	1	2533.194	89.917	0.000	0.552
	OLE Post	7167.132	1	7167.132	46.535	0.000	0.389
Error	EV Post	969.716	73	13.284			
	IDLE Post	6440.633	73	88.228			
	Techno Post	2056.609	73	28.173			
	OLE Post	11243.107	73	154.015			
Total	EV Post	92000.000	79				
	IDLE Post	140592.000	79				
	Techno Post	67593.000	79				
	OLE Post	175443.000	79				
Corrected Total	EV Post	3910.835	78				
	IDLE Post	16897.367	78				
	Techno Post	6382.861	78				
	OLE Post	19225.671	78				

It can be seen from Table 3 that the treatment (learning and practicing vocabulary using a social website on Instagram) has a statistically significant effect on EV (*F* (1, 73) = 59.644; *p* < .05; partial η^2^ = 0.450), IDLE (*F* (1, 73) = 83.556; *p* < .05; partial η^2^ = 0.534), Technostress (*F* (1, 73) = 89.917; *p* < .05; partial η^2^ = 0.552), and OLE (*F* (1, 73) = 45.535; *p* < .05; partial η^2^ = 0.389). Thus, it can be easily concluded that the EG’s vocabulary learning, informal digital learning of English, and on-line engagement improved significantly and their technostress decreased accordingly.

## Discussion

The objective of this study was to demonstrate the importance of appropriate MALL apps in the process of learning English, specifically focusing on the acquisition of English vocabulary using Instagram feed-based assignments. Utilizing Instagram activities that are tailored to user feeds seems to have a beneficial influence on students’ comprehension of English vocabulary. In other words, the students in the experimental group surpassed their counterparts in the control group. The results underscore the auspicious potential of MALL in the instruction and acquisition of the English language via the use of Instagram. The study’s findings provide insights into the potential benefits of using Instagram feed-based assignments to enhance vocabulary instruction.

It is possible that students will be more completely engaged in the learning activities if they are presented with a variety of unique and creative task suggestions on Instagram. This may be compared to the control group that was used in this study. The usefulness of Instagram as a MALL tool within the context of enhancing students’ motivation, engagement, and viewpoint on education has been shown by previous studies [1, 5, 36, 38, 39, 40] Asserted that MALL helps students acquire new words while freeing up class time for other objectives. This is similar to what was said in the previous paragraph. EFL students could gain something by utilizing their mobile devices for communication outside of the classroom, according to [41]. Additionally [2], suggested that the use of social media has a significant role in assisting students in being more self-aware and introspective as they learn a second language.

Instagram has the potential to use images, music, and videos to impart vocabulary wisdom. As was previously said, we planned to use images, music, and video on our Instagram profile to impart grammatical wisdom. The students’ enthusiastic responses and obvious engagement with the lessons show they appreciated the audio and video explanations of grammar concepts. One possible justification for this is the idea that engaging students’ aural and visual senses leads to more engaged learning. [4] investigated the topic of video-based grammar education and came to the same conclusion: it got pupils actively engaged in the learning process. Additionally, it was shown that exercises based on Instagram feeds are beneficial for learning grammar and may influence the attitude of EFL learners [7, 42, 43].

Also shown by [44, 45] is the fact that Instagram offers students of English as a foreign language a fantastic opportunity to practice and enhance their language abilities. In the same line of investigation [46], employed MALL to teach Taiwanese students of English as a foreign language speaking exercises. According to the findings of their study, students who utilize MALL to completely immerse themselves in their curriculum may see an enhancement in their ability to communicate effectively in front of others. In addition [47], used the mobile application Kahoot! as a teaching tool in order to enhance the vocabulary learning practices of their pupils. They made the discovery that the use of media with the purpose of teaching vocabulary, such as movies and music, stimulated participatory learning. It is clear that the students valued the audio and video explanations of vocabulary topics because of their passionate reactions and their apparent involvement with the lectures. A potential rationale for this is the notion that stimulating the auditory and visual senses of pupils results in increased levels of engagement in the learning process. In addition [48], discovered that learners’ buoyancy, mood control, and anxiety management were improved with the use of telegram in online instruction.

Additionally, it was demonstrated that students’ level of commitment to their online classes increased when they participated in IDLE events. Students were able to go beyond what was taught in the classroom by participating in non-formal activities to improve their language abilities. Some examples of these activities include playing online games that focused on language and watching movies in English. The learner’s experience with formal language instruction was supplemented and extended by these IDLE tasks, which provided the learner with important exposure to and practice in the target language. In light of this greater familiarity with the language, students took a more proactive part in their online learning.

The findings of the current study demonstrated that IDLE had a highly predictive role in predicting the degree to which students actively engage in online courses, and that the social presence of the students acts as a mediator between themselves and this link. According to the findings of other studies, most notably [4], who discovered a similar positive impact of IDLE on students’ social presence, our findings are in agreement with their findings. The suite of applications and tools that IDLE provides has been shown to have a substantial influence on the capacity of students to engage in conversation with one another and learn from one another in virtual environments [7, 33]. Activities like as the usage of language-focused applications, the watching of movies linked to the target language, and active participation in language exchanges across various social media platforms are all examples of activities that may be done in the target language. These extracurricular activities push language learning beyond the confines of the conventional online classroom [49] and give students with essential opportunities for complete immersion in the language that they are studying.

Along with that, the findings have demonstrated that the adoption of a practical approach to the acquisition of vocabulary not only motivates students to take part in their own assessments, but also lessens the amount of anxiety that students feel while they are actively participating in online classes. According to the findings of this study, maintaining a healthy emotional and cognitive equilibrium is necessary in order to effectively manage the effects of technological stress. In addition to providing EFL students with an insider’s perspective on their capabilities and limits, the results of this research show that the use of a user-friendly application is essential for the management of technological stress. Instagram provides students with greater latitude in their work, which enables them to create and strengthen their higher-order thinking abilities. Additionally, Instagram allows students to pick meta-cognitive techniques, which is another probable reason for the outcomes that were achieved. Thus, EFL students are provided with the opportunity to consider themselves not just as passive learners, but also as distinct individuals with their own preferences and needs for completing and improving their studies.

It is possible that more empirical research might be beneficial to this topic, which seems to be in its infancy, by lighting a path that enhances the academic performance of students and assures efficient education. It is essential for decision-makers, curriculum designers, content producers, test creators, and language instructors to realize the benefits of integrating psychological aspects that have the ability to lessen the stress that children may feel while taking a language assessment. Work that teaches students of English as a foreign language how to use helpful self-help frameworks outside of the classroom is also welcomed. When learning a language, it is important to develop abilities such as self-regulation and self-awareness from the very beginning, particularly when participating in online training. As a result, EFL students will have a higher chance of succeeding with technology, teachers will be able to better tailor their teaching to the individual learners they are dealing with, and everyone will benefit.

The findings that correspond to the third study question demonstrated that students of who used Instagram exhibited improved levels of participation and motivation. The results of this study are consistent with the findings of [20], which discovered that encouraging students to participate in self-evaluation leads to an improvement in the students’ self-control and confidence, which in turn leads to an increase in the students’ enjoyment of the procedure of learning. In a comparable manner [24] found that participation in online assessment led to increased levels of resilience and autonomy among EFL students. On the basis of theoretical considerations, this conclusion may be contested. The principle of self-determination and the concept of maintaining one’s own autonomy [50, 51]. For EFL students, an instruction strategy that is more learner-centered may offer both obvious and covert advantages. According to the concepts of Connectivism, which emphasized the importance of online networks and social media for skill learning, the findings may be theoretically validated. Due to the increased time spent immersed in the target language, students taking classes online have a better chance of reaching a higher level of proficiency. Researching the effects of IDLE on students’ drive and engagement in online classes therefore has theoretical backing.

## Conclusion

Overall, this research aimed to add to the limited understanding of how Instagram feed-based assignments might enhance vocabular learning among intermediate EFL learners. The analysis of the findings indicated that state of IDLE, Technostress, and engagement of the learners may change due to applying suitable application in language learning. The results of this research indicate many pedagogical consequences for learners, instructors, and the educational system. It is crucial to acknowledge that integrating technology (e.g., Instagram) into curricula may significantly enhance learner’s accessibility to course information and provide possibilities for engagement with the course outside the classroom. Furthermore, it has been shown that MALL is a beneficial feature for acquiring and honing grammatical skills. This is because learners are actively involved in generating or consuming educational material using a device that is easily accessible and of their preference. A further aspect that warrants particular consideration is the distinct role of educators in implementing Instagram feed-based assignments inside L2 courses. Proficiency in L2 digital literacy is essential for effectively using social media. Therefore, it is recommended that language instructors get comprehensive training on this significant aspect as part of their pre- and in-service teacher education programs. To clarify, it is essential for language instructors to have training that enables them to use social media with proficiency. By adopting this approach, the learning environment will benefit from more effective education.

Future studies may want to dig more into some of the questions raised by the study’s caveats. These findings have limited use because of the quasi-experimental character of the subjects chosen. The effects of applying different apps on informal digital learning of English, technostress, on-line engagement, and language achievement need to be studied over time. In addition, demographic considerations were not taken into account. Future research of this kind will benefit greatly from the addition of instructor demographic data. Although this study utilized a quantitative methodology, it’s possible that a mixed-methods inquiry may provide superior outcomes. Last but not least, the relationship between informal digital learning of English, technostress, on-line engagement, language achievement, as well as other learner attributed qualities including buoyancy, readiness to speak, and L2 grit might be the focus of future research.

### Electronic supplementary material

Below is the link to the electronic supplementary material.

**Additional File 1: Appendix A:** Oxford Quick Placement Test

**Additional File 2: Appendix B:** Vocabulary test

## Data Availability

The dataset of the present study is available upon request from the corresponding author.

## Declarations

Written informed consent to participate in this study was provided by the participants.

All the experiments in our study were conducted in accordance to the relevant guidelines and regulations of 1963 Helsinki declaration and its later amendments.

## Abbreviations

EFL: English as a Foreign Language Learner
MANOVA: Multivariate Analysis of Variance
EG: Experimental Group
CG: Control Group
MALL: Mobile Assisted Language Learning
CALL: Computer Assisted Language Learning
IDLE: Informal Digital Learning of English
OQPT: Oxford Quick Placement Test
IDLES: Informal Digital Learning of English Scale
T-S S: Techno-stress Scale
BE: Behavioral Engagement
CE: Cognitive Engagement
AE: Affective Engagement
SE: Social Engagement
KST: Kolmogorov–Smirnov test
EV: English Vocabulary
OLE: On-Line Engagement
