# Supplementary material for: Wow, I cannot stop: a concentration on vocabulary learning via instagram and its effects on informal digital learning of english, technostress, and on-line engagement
Source: BMC Psychol. 2024 Jan 2;12:8. doi: 10.1186/s40359-023-01503-w (PMC10759675; doi:10.1186/s40359-023-01503-w)
Supplement: Supplementary file 2 — Additional File 2: Appendix B: Vocabulary test [file 40359_2023_1503_MOESM2_ESM.docx]

**Appendix B:**

**Vocabulary Test**

1. I like Manchester team. It is my ……………..team.

a. good b. excellent c. perfect d. favorite

2. I put out my coat since the weather was…………

a. rainy b. sunny c. windy d. cold

3. he can………………on the card.

a. speak b. write c. read d. write

4. My little brother has straight hair. His hair is not……………..

a. long b. short c. curly d. dry

5. Millions of different plants and animals live in tropical……………

a. buildings b. homes c. rainforests d. parks

6. Plants and trees like sunlight and rain, so they …………fast.

a. die b. grow c. develop d. decrease

7. Monkeys…………….. from tree to tree.

a. jump b. fly c. run d. walk

8………………study dinosaur bones to find out how they lives and what they liked like.

a. teachers b. students c. practitioners d. scientists

9. Usain Bolt is a runner and won three gold ……………at the Olympics.

a. medals b. books c. shoes d. balls

10. This African language has a few ............... .

a. information b. speakers c. dictionary d. news

11. It is really hard to understand Chinese. “Hard” is closest in meaning to: ............... .

a. lucky b. difficult c. real d. amazing

12. The number of endangered languages is largely increasing. “Largely” is closest in meaning to ............ .

a. nearly b. really c. greatly d. probably

13. We collect food and ............... clothing for poor people.

a. next b. probable c. extra d. honest

14. I always wish to have a ………. of cake with my coffee.

a. bar b. slice c. tube d. loaf

15. Education and training are the most effective ………. of improving the nation’s economy.

a. cultures b. means c. institutes d. skills

16. As I ………. earlier, sales this year have been lower than expected.

a. existed b. mentioned c. increased d. respected

17. The laws suggest that relationships between members of ………. are organized in terms of rules.

a. destination b. society c. culture d. knowledge

18. They began ………. the area carefully, taking in every detail and watching for the slightest movement.

a. measuring b. predicting c. scanning d. destroying

19. She said the letter had caused her ………. hurt and distress at a very difficult time.

a. additional b. favorite c. cultural d. popular

20. Sometimes you have to remind yourself that most of your problems only … in your head.

a. invite b. interest c. exist d. disappear

**2**1. Monkeys…………….. from tree to tree.

a. jump b. fly c. run d. walk

22. I put out my coat since the weather was…………

a. windy b. rainy c. sunny d. cold

23. Sometimes you have to remind yourself that most of your problems only … in your head.

a. invite b. interest c. exist d. disappear

24. My little brother has straight hair. His hair is not……………..

a. curly b. short c. long d. dry

25. Millions of different plants and animals live in tropical……………

a. buildings b. homes c. rainforests d. parks

26. Plants and trees like sunlight and rain, so they …………fast.

a. die b. grow c. develop d. decrease

27. I like Manchester team. It is my ……………..team.

a. favorite b. excellent c. perfect d. good

28………………study dinosaur bones to find out how they lives and what they liked like.

a. teachers b. students c. practitioners d. scientists

29. Usain Bolt is a runner and won three gold ……………at the Olympics.

a. medals b. books c. shoes d. balls

30. This African language has a few ............... .

a. information b. speakers c. dictionary d. news

31. It is really hard to understand Chinese. “Hard” is closest in meaning to: ............... .

a. lucky b. difficult c. real d. amazing

32. The number of endangered languages is largely increasing. “Largely” is closest in meaning to ............ .

a. nearly b. really c. greatly d. probably

33. We collect food and ............... clothing for poor people.

a. next b. probable c. extra d. honest

34. I always wish to have a ………. of cake with my coffee.

a. bar b. slice c. tube d. loaf

35. Education and training are the most effective ………. of improving the nation’s economy.

a. cultures b. means c. institutes d. skills

36. As I ………. earlier, sales this year have been lower than expected.

a. existed b. mentioned c. increased d. respected

37. The laws suggest that relationships between members of ………. are organized in terms of rules.

a. destination b. society c. culture d. knowledge

38. They began ………. the area carefully, taking in every detail and watching for the slightest movement.

a. measuring b. predicting c. scanning d. destroying

39. She said the letter had caused her ………. hurt and distress at a very difficult time.

a. additional b. favorite c. cultural d. popular

40. He can………………on the card.

a. speak b. write c. read d. write
